# Technological Proficiencies, Engagement, and Practical Considerations for mHealth Programs at an Urban Safety-Net Hospital Emergency Departments: Data Analysis

**DOI:** 10.2196/23641

**Published:** 2022-06-06

**Authors:** Sean Treacy-Abarca, Janisse Mercado, Jorge Serrano, Jennifer Gonzalez, Michael Menchine, Sanjay Arora, Shinyi Wu, Elizabeth Burner

**Affiliations:** 1 David Geffen School of Medicine University of California Los Angeles Los Angeles, CA United States; 2 Keck School of Medicine University of Southern California Los Angeles, CA United States; 3 University of Southern California Los Angeles, CA United States; 4 Schaeffer Center for Health Policy and Economics University of Southern California Los Angeles, CA United States; 5 Department of Emergency Medicine Keck School of Medicine University of Southern California Los Angeles, CA United States; 6 Suzanne Dworak-Peck School of Social Work University of Southern California Los Angeles, CA United States; 7 Viterbi School of Engineering University of Southern California Los Angeles, CA United States; 8 Department of Preventative Medicine Keck School of Medicine University of Southern California Los Angeles, CA United States

**Keywords:** mHealth, engagement, practical considerations, safety-net hospital, emergency department, minority health, low income

## Abstract

**Background:**

Safety-net emergency departments often serve as the primary entry point for medical care for low income predominantly minority patient populations. Herein, we sought to provide insight into the feasibility, technological proficiencies, engagement characteristics, and practical considerations for a mHealth intervention at a safety-net emergency department.

**Objective:**

We aimed to analyze patient technological proficiency to understand the feasibility of and draw practical considerations for mobile phone technology (mHealth) solutions for patients with chronic disease served by safety-net emergency departments.

**Methods:**

We analyzed data from a previous diabetes randomized clinical mHealth trial for a diabetes social support intervention. Patients from a safety-net emergency department with preexisting diabetes who used SMS text messages, owned a mobile phone, and with hemoglobin A_1c_ levels >8.5% were enrolled. A text message–based mHealth program to improve disease self-management was provided to all patients. Supporters of patients were randomized to receive a mailed copy or mHealth-based curriculum designed to improve diabetes support. Among enrolled patients, we surveyed mobile technological capacity and frequency of use. We performed latent class analysis to identify classes of patients by level of technological proficiency and compared demographic characteristics between the latent classes to identify demographic subgroups that may require more training or tailoring of the mHealth approach. Study engagement between classes was assessed by comparing the mean number of text messages exchanged, loss to follow-up, and early termination.

**Results:**

Of 1876 patients who were approached, 44.2% (n=829) of patients had a stable mobile phone and were able to use text messages. Among them 166 met the trial inclusion and enrolled, 90% (149/166) of the cohort were ethnically diverse. Significant variance was found in technology capacity and frequency of use. Our latent class analysis classified 75% (124/166) of patients as highly technologically proficient and 25% (42/166) patients as minimally technologically proficient. Age (*P*<.001) and level of education (*P*<.001) were associated with class membership. Highly technologically proficient patients were younger and had higher levels of education (45.74 years old; high school or more: 90%) than minimally technologically proficient patients (53.64 years old; high school or more: 18%). Highly technologically proficient participants exchanged a mean of 40 text messages with the system coordinators compared to a mean of 10 text messages by minimally technologically proficient patients (*P*<.001).

**Conclusions:**

This study found that nearly half of the patients screened at the safety-net emergency department were equipped for an SMS text message–based mHealth intervention. In the small sample of patients who were enrolled, the majority were classified as highly technologically proficient. These highly proficient patients had greater study engagement. mHealth use in emergency departments may be an opportunity to improve health of ethnically diverse populations by pairing sophisticated chronic disease self-management program with SMS text message–based and traditional in-person interventions to reach patients through the method that is most familiar and comfortable.

**International Registered Report Identifier (IRRID):**

RR2-10.1016/j.cct.2019.03.003

## Introduction

Over the past two decades, mobile phone technology interventions for health (mHealth) have expanded rapidly into most specialties, settings, and patient populations [1,2]. mHealth interventions have been used to improve self-management for a spectrum of chronic diseases, including hypertension, diabetes, and colon cancer. mHealth interventions have recently and successfully used reminders, feedback, and planning prompts to improve health care utilization and improve self-care of chronic illness [3-6]. The strength of mHealth interventions lies in their low financial costs and minimal requirements for additional human capital or new infrastructure [7]. However, the adoption of mHealth interventions has lagged in vulnerable populations such as low-income groups, racial and ethnic minorities, and underinsured or uninsured populations [1,8].

Descriptions of mHealth implementations for vulnerable populations in the United States are limited. Despite limited adoption of mHealth interventions in low-income Latino populations, preliminary studies highlight the missed opportunity to improve chronic disease self-management [9-11]. In these populations, mHealth has improved medication adherence, diabetes control, and health care utilization patterns and has reduced health care costs [12]. These benefits are evident regardless of a patient’s baseline health literacy [7,13]. mHealth interventions have been shown to improve the care of minority and underserved Latino and African American patient populations [8]. The appropriate setting and the fundamental characteristics of mHealth implementation in Latino communities have not been fully explored as most interventions have been deployed in specialty clinics or with patients with existing access to primary care.

Safety-net health systems, which are charged with providing preventative and advance health services to those with limited ability to pay, have been considered for mHealth solutions, but a fundamental understanding of appropriate communication modalities is needed, which has hindered mHealth updates in this venue. Approaches to ensuring appropriate mHealth solutions deployed in safety-net health systems include the use of small focus groups [14]. Safety-net health system outpatient community care clinics with younger and more adequately insured patients with higher levels of socioeconomic standing are well equipped for mHealth solutions [15]. Although nearly 16,270 papers have been published in the mHealth arena, only 16 papers contributed knowledge on behalf of historically underserved and minority populations [16]. Understudied minority patient populations have potential for mHealth success—successful mHealth strategies have been conducted in African American and Korean populations—including the delivery of HIV prevention strategies to black youth, mobile phone–based counseling among pregnant teen mothers, cervical cancer screening among Korean American women [17-19]. However, the limits of mHealth solutions in more clinical situations must be explored—most notably, in safety-net emergency departments primarily serving minority and medically underserved patients.

Emergency departments are underutilized settings for mHealth solutions despite their increasing role in chronic disease management. The role of emergency departments in acute and chronic care for low-income minority patients continues to evolve under the Affordable Care Act [14,15]. This group disproportionately uses safety-net emergency departments as a primary entry point to the medical system [16-18]. The Agency for Health care Research and Quality estimates that safety-net hospitals account for roughly 25% of all hospitals yet over 33% of all in-patient stays in the United States [19]. This intersection between low-income minority patient populations and their entry to medical care makes emergency departments a promising location for mHealth intervention strategies for chronic diseases. Additionally, mHealth solutions can provide asynchronous patient education and care, which is well suited for emergency departments given that providers may not have sufficient time to engage patients in lasting behavior change in the pressured environment of the emergency department.

Safety-net emergency departments may be an excellent setting for mHealth interventions, but information on large-scale feasibility and implementation for heterogenous patient populations is limited in the current literature. While mHealth implementation among minority and historically underserved patient populations has been documented, we do not have a full understanding of fundamental tenants of mHealth implementation for the diverse patients presenting to safety-net emergency departments in the United States. In the few reported studies, emergency department–based mHealth interventions have decreased overall emergency department utilization [20], improved patient knowledge on safe opioid use [15], and have been feasible in management of alcohol use disorder [11]. Additionally, complex determinants of health, such as social support, can improve with the use of an mHealth platform by patients in the emergency department [21]. Despite these initial positive results, we do not know how technological proficiencies may affect engagement with mHealth solutions in populations using safety-net emergency departments or if patients with chronic diseases who use safety-net emergency departments are ready to engage with mHealth. mHealth trials conducted within minority, low socioeconomic status, and underserved populations have traditionally successfully recruited 13% to 40% of approached patients [22]. We have limited insight into the feasibility of mHealth utilization in busy safety-net emergency departments or into which approaches can be used to improve enrollment and the use of mHealth. National estimates do not accurately reflect the mobile technology use of underserved patients who present to these emergency departments [22]. Additionally, patients who visit emergency departments have less technology proficiency, such as lower app utilization rate compared to national estimates [23]. The modalities used by current mHealth solutions may not be congruent with the technological capacities of the understudied safety-net emergency department population.

Herein, we examine the feasibility of deploying mHealth solutions at a busy safety-net emergency department and describe the technological proficiency of a diverse cohort of patients with diabetes presenting to a safety-net emergency department by using data from a pragmatic mHealth randomized clinical trial on a text message–based social support intervention. We asked (1) are diverse safety-net emergency department patients equipped for mHealth interventions, (2) does diversity of patient demographics determine technological proficiency, and (3) is technological proficiency associated with mHealth engagement?

## Methods

### Ethics Approval

The study was approved by the Health Sciences Campus of University of Southern California Institutional Review Board (approval number HS-17-00406).

### Study Population and Design

We used data from the TExT-MED+FANS mHealth randomized clinical trial [10]. The 6-month randomized clinical trial was conducted to understand the role of social support in improving chronic disease self-management by using an SMS text message–based bidirectional mHealth platform in the patient’s choice of language (English or Spanish). All patients enrolled in the study were given access to the mHealth program [24] and asked to identify a supporter upon enrollment. A supporter was broadly defined as a patient’s acquaintance, family, or spouse who shares the common goal of improving the well-being of the patient. Supporters of these patients were randomized to a mHealth intervention designed to improve diabetes specific support or to receive identical information provided as printed material in a pamphlet control. Patients were recruited from the Los Angeles County–University of South California Medical Center safety-net emergency department, which serves predominantly an ethnic minority (Latino ethnicity) patient population, with 132 beds and over 150,000 annual visits [23], was conducted from July 2017 to October 2018.

### Study Procedures

Trained research assistants screened patients via a real-time emergency department electronic tracking board and health records for diabetes. Patients were ineligible to participate if they had hemoglobin A_1c_ levels less than 8.5%, could not identify a supporter, or denied having diabetes. Technology inclusion criteria were met if participants could send and receive text messages and had access to a mobile phone for more than 30 days.

Patients’ self-reports of technological proficiency were collected at study enrollment, upon initial presentation to the emergency department. Information was collected via the Mobile Usage Survey derived from the Pew National Survey of Latinos to understand the capacity and frequency of respective technology use habits [2]. Demographic information included age, race, ethnicity, gender, primary language, English proficiency, and country of birth. Technological proficiency characteristics included access the internet and the ability and frequency to send or receive SMS text messages, send and receive instant messages, send and receive emails, and to use mobile phone apps. mHealth platform engagement metrics were assessed using data furnished by the third-party provider of the mHealth platform (Agile Health). Engagement metrics included the mean number of SMS text messages exchanged with the mHealth platform, study dropout rate, and requests for program termination.

### Statistical Analysis

We used Stata (version 16; StataCorp LLC) for data analysis. Statistical differences were ascertained using chi-square tests. *P* values<.05 were statistically significant. Using data from the technological proficiency survey, we stratified study participants into an undefined number of underlying subgroups using latent class analysis. Prior studies [25] have employed latent class analysis to better understand subgroups of patients such as high utilizers of safety-net hospitals. Latent class analysis is a person-centered, finite mixture model technique which sorts survey participants into latent classes using observable variables, using probabilistic distribution functions with the goal of establishing the most parsimonious and interpretable set of classes [26]. We used M-plus (version 1.6; Muthén & Muthén) to fit a latent class model. We evaluated an increasing number of classes starting at a 1-class solution, using the following manifest variables: ability to send and receive SMS text messages, send and receive emails, access the internet, send and receive instant messages, or ability to use apps. Class size was increased by 1 in each sequential analysis and measures of fit (Akaike information criterion, Bayesian information criterion, and entropy) were examined at each step. Analysis continued sequentially until at least 2 sequential models showed a poorer fit than the best prior model. The 2-class model was found to have the highest entropy and lowest Akaike information criterion and Bayesian information criterion–penalized likelihood criteria.

The manifest variables defining technological proficiency were examined to describe the 2 classes—highly technologically proficient or minimally technologically proficient. Demographic differences between the 2 classes were assessed using independent 2-tailed *t* test and chi-square test *P* values. Information derived from the technology survey and latent class analysis was used to understand if baseline technological proficiency characteristics were associated with study engagement. *Texts exchanged* with study coordinators were compared using the 2-sample *t* test. Additionally, we assessed differences in study dropout rate as well as SMS text message termination requests between the classes of participants using nonparametric tests.

## Results

### Feasibility of Screening and Recruitment

**Figure 1:**
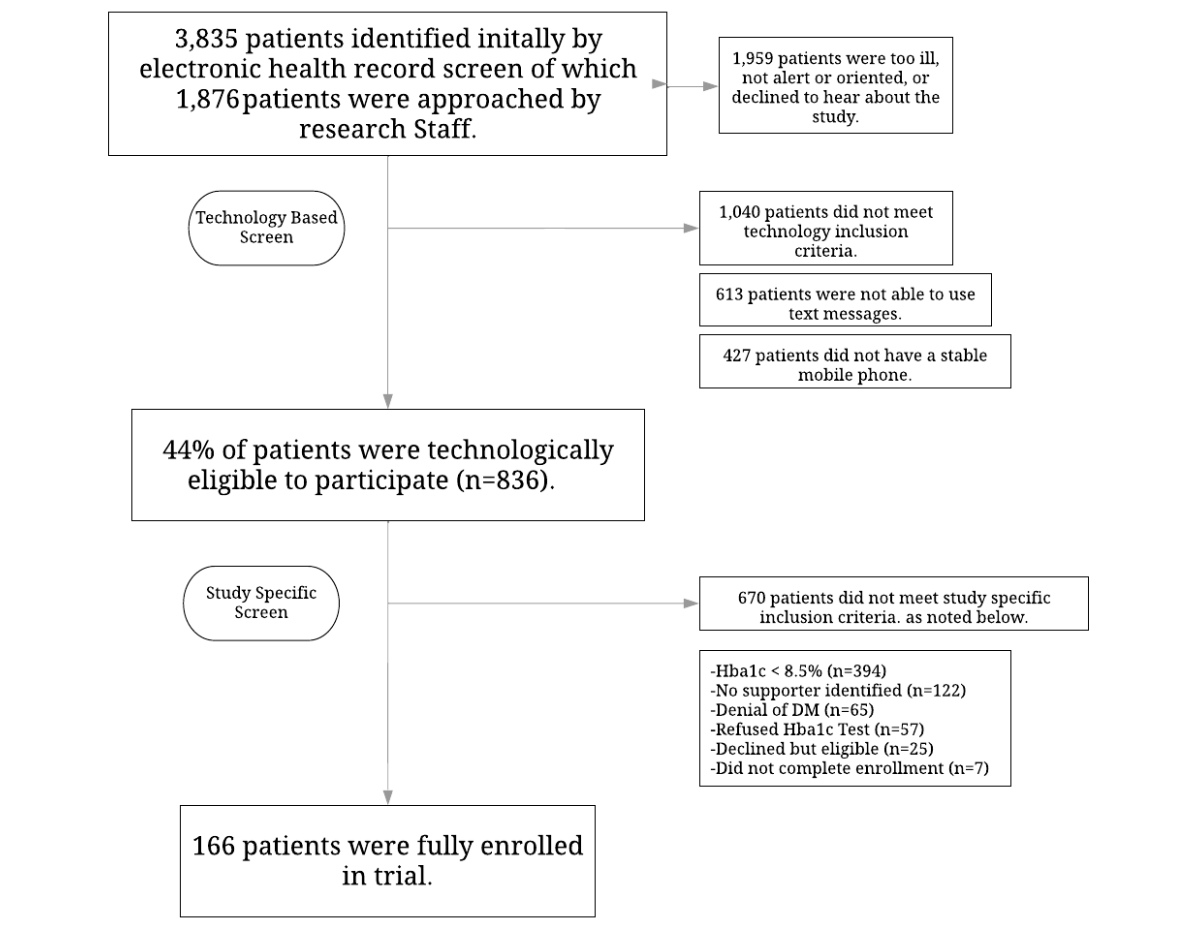
Recruitment diagram. DM: diabetes; HbA_1c_: hemoglobin A_1c_.

In total, 3835 emergency department patients were screened: 1959 patients were not approached due to a variety of factors including the patient’s severity of illness, the patient was not alert or oriented, the patient declined to hear about the study, or the patient was discharged before research staff could approach them (Figure 1). Of the 1876 patients approached, we successfully identified 44.2% (n=829) of patients as owning a mobile phone and capable of sending and receiving text messages. This shows the safety-net emergency department patients, who are primarily of Latino ethnicity, are equipped for mHealth interventions.

An additional 670 patients were ineligible because hemoglobin A_1c_ levels were <8.5% (n=394), no supporter was identified (n=122), they denied having diabetes (n=65), or they declined to participate (n=25). Moreover, 7 patients did not complete enrollment on mHealth platform. Thus, 166 patients were fully enrolled into the study, and all received access to the mHealth platform.

Of 166 enrolled patients, 82 (49.4%) were male, and 84 (50.6%) were female; none selected nonbinary. Most patients identified as racial and ethnic minorities (Table 1). The majority of participants were foreign born Mexico: 131 (78.7%) patients were born in Mexico or Central America, and 116 (69.9%) patients preferred Spanish. All patients owned mobile phones, and 5% (8166) reported sharing their phone with another family member. Patients had a variety of mobile phone service plans: 33.7% of patients (56/166) reported having a contract-based mobile phone, 53% of patients (88/166) paid per month, and 13% of patients (22/166) had some other type of payment arrangement.

**Table 1:** Participant demographic data.

Characteristic			Value (n=166), n (%)
**Gender**			
	Male	82 (49.4)	
	Female	84 (50.6)	
	Nonbinary	0 (0)	
**Ethnicity**			
	Hispanic or Latino	156 (94.0)	
	Not Hispanic or Latino	9 (5.4)	
	Unknown or not reported	1 (0.6)	
**Race**			
	American Indian/Alaskan Native	5 (3.0)	
	Asian	2 (1.2)	
	Black or African American	9 (5.4)	
	White	74 (44.8)	
	Mixed	3 (1.8)	
	Unknown or not reported	72 (43.6)	
**Country of birth**			
	Mexico	100 (61.0)	
	United States	35 (21.3)	
	El Salvador	11(6.7)	
	Other	18 (11.0)	
**Language preference**			
	English	50 (30.1)	
	Spanish	116 (69.9)	
**Level of education**			
	No formal education	4 (2.4)	
	Grammar	53 (32.1)	
	High school	77 (46.7)	
	College or vocational	29 (17.6)	
	Professional	2 (1.2)	

### Latent Class Analysis of Study Participants’ Technology Use

**Figure 2:**
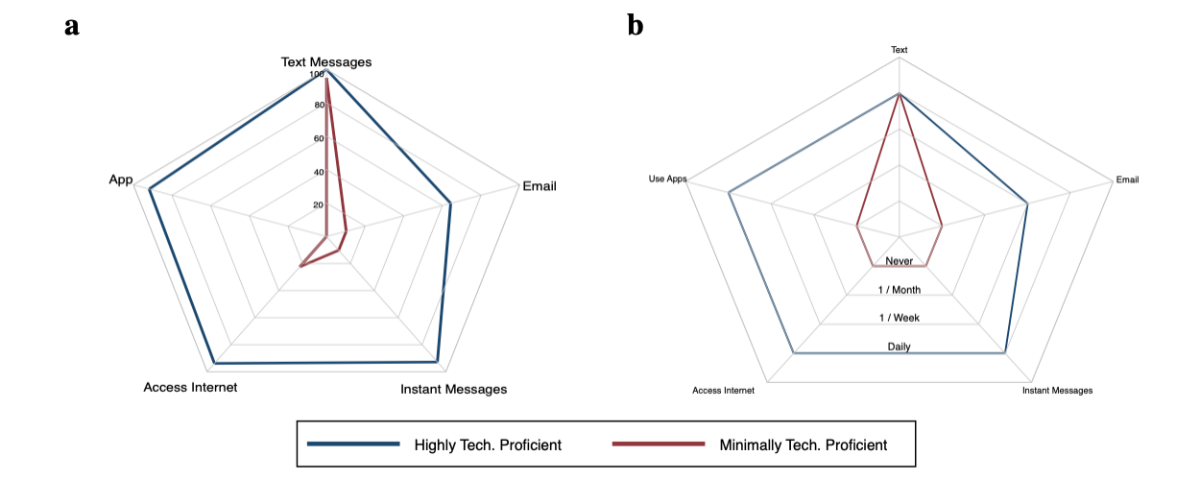
Baseline (a) technological capacity and (b) frequency of use of mobile phone by patient class.

In the latent class analysis, a 2-class model best identified underlying classes with 75% (124/166) of patients classified as highly technologically proficient and 25% (42/166) of patients as minimally technologically proficient. The 2 classes differed in technology capacity and frequency of technology use (Figure 2), except for SMS text message capability. All highly technologically proficient patients and 95% of the minimally technologically proficient patients used SMS text messages. Compared with minimally technologically proficient patients, highly technologically proficient patients were more likely to use email (65% vs 12%, *P*<.001), use instant messages (93% vs 12%, *P*<.001), use apps (92% vs 2%, *P*<.001), and access the internet (94% vs 24%, *P*<.001). Similarly, highly technologically proficient patients more frequently reported daily use of SMS text messaging (87% vs 55%, *P*<.001), email (46% vs 5%, *P*<.001), instant messages (73% vs 5%, *P*<.001), app use (81% vs none, *P*<.001), and internet use (78% vs 10%, *P*<.001).

We also compared latent class differences in demographic characteristics of race, ethnicity, age, language proficiency, and country of birth between classes. No differences between patient classes were found for gender (*P*=.17), race (*P*=.62), ethnicity (*P*=.82), country of birth (*P*=.50), or language preference (*P*=.07) (Table 2). However, there were differences between the 2 patient classes in level of education (*P*<.001) and mean age (*P*<.001) (Table 2). Highly technologically proficient patients’ mean age was 45.74 years compared to 52.73 years for minimally technologically proficient patients (*P*<.001). Lastly, highly technologically proficient patients had statistically significant (*P*=.05) greater engagement with the mHealth platform, exchanging an average of 40.94 exchanged SMS text messages through the 6-month study duration compared to 10.79 SMS text messages for minimally technologically proficient patients (Table 2). No statistical differences (*P*=.85) in study loss to follow-up or request for SMS text message curriculum termination were noted between the 2 patient classes.

**Table 2:** Demographic and study participation measures by patient class.

Demographic measures			Highly proficient (n=124)		Minimally proficient (n=42)		*P* value	
**Gender, n**								.17
	Female	59		25				
	Male	65		17				
Age (years)			45.74		52.73		<.001	
**Ethnicity, n**								.82
	Hispanic or Latino	116		40				
	Not Hispanic or Latino	7		2				
	Unknown	1		0				
**Race, n**							.62	
	American Indian/Alaska Native	3		1				
	Asian	2		0				
	Black or African American	6		3				
	White	58		16				
	Mixed	3		0				
	Unknown or not reported	52		22				
**Country of birth, n**							.50	
	United States	28		7				
	Mexico	74		28				
	El Salvador	7		4				
	Other	15		3				
**Language preference, n**							.07	
	English	42		8				
	Spanish	82		34				
	Other	0		0				
**Education level, n**							<.001	
	No formal education	3		1				
	Grammar	29		23				
	High school	62		17				
	College or vocational	28		1				
	Professional school	2		0				
**Engagement measures**								
	Text messages, mean	40.94		10.79		.05		
	6-month loss to follow-up, %	41.46		39.53		.82		
	Early text message termination, %	7.32		2.33		.24		

## Discussion

### Principal Findings

Most emergency department patients with diabetes who could engage in mHealth were highly technologically proficient. We found that age and level of education differed between highly and minimally technologically proficient counterparts but that other demographic characteristics did not differ between classes. We provide practical suggestions for designers planning to expand mHealth use among the diverse patient populations served at safety-net emergency departments (Table 3).

Safety-net emergency departments are feasible clinical settings for mHealth solutions for underserved patient populations, with a good portion of patients having sufficient capacity to engage: 44.2% of patients (829/1876) that we approached in the emergency department fulfilled basic technology-based eligibility criteria. A cohort of users was recruited and enrolled despite the acuity and fast paced environment of the emergency department. Our success here is consistent with those of other mHealth solutions provided to minority, low income, and underserved patient populations from other clinical settings, which have reported eligibility success between 13% to 41% [22,23,27]. A particular strength of our study is the racial and ethnic diversity of our cohort with respect to the general population that have been traditionally studied for mHealth interventions. We found that only age and education influenced technological capacity, in line with the findings of a previous study [8] that both age and indicators of socioeconomic status, such as education, were associated with improved mHealth solution uptake. Gender, ethnicity, race, country of birth, and language were not determinants of highly technologically proficient status. Future researchers on mHealth solutions at safety-net emergency departments should take into consideration both patient age and education when designing mHealth interventions, but a diverse patient cohort is not an impediment toward successful mHealth solutions at safety-net emergency departments.

**Table 3:** Findings and practical considerations for mHealth interventions for chronic diseases in safety-net emergency departments.

Finding	Implication
Safety-net emergency departments allow for recruitment of diverse patient cohorts	Recruitment for mHealth intervention trials is feasible among across culturally, linguistically, racially or ethnically, and geographically diverse populations.
Optimal mHealth technological modalities exist in safety-net emergency department patients	Future designs should consider text message–based interventions as a primary modality, as well as instant message–based modalities, and app-based modalities.
Less optimal mHealth technological modalities exist in this diverse study cohort	Email-based mHealth interventions are particularly poorly suited as email was used the least by either patient classification, and serial surveys of technological proficiency should be conducted as capacities evolve and new technology becomes available.
Most demographic characteristics are not associated with to highly technologically proficient classification	In our study population, gender, ethnicity, race, country of birth, or language preference were not associated with classification as highly technologically proficient and should not be used for mHealth intervention eligibility.
Age and level of education are associated with highly technologically proficient classification	Additional research is needed to understand to how to harness this finding for improvement in clinical outcomes, and differential design of studies.
Highly technologically proficient patients had greater mHealth engagement	Future studies should be conducted to improve engagement among minimally technologically proficient patients and to understand the costs and benefits of targeted training for patients to improve engagement.

While safety-net emergency departments are feasible settings for mHealth, we must carefully consider which technological modalities to deploy. Text messages, web-based, instant message–based, and apps may be well suited modalities for safety-net hospital systems if age and educational considerations are built into the design of the interventions. Interestingly, email-based capacity for use and frequency of use were low for both classes. The finding of limited email use is a departure from mHealth solutions conducted at clinical settings that are not safety-net hospitals [3]. Visits to safety-net emergency departments by minority and historically underserved patient populations can be capitalized on by expanding mHealth solutions as emergency departments serve as primary entry points to primary care and specialty care [18]. Such expansion can be aided by considering suitable technological modalities, technological capacity within this population, drivers of technological capacity, and effect on engagement with mHealth solutions. We found that highly technologically proficient patients had better engagement with the mHealth platform, understanding how to increase engagement in low-resource settings may be critical to successful interventions [28].

As the field of mHealth expands into resource-limited settings and participants, our study presents unique insights into patients that could be targeted for optimization of mHealth solutions and increasing patient engagement. The minimally technologically proficient cohort lacked robust use of most technological modalities beyond text messages. The communication modality selected for mHealth interventions in safety-net emergency departments may need to be congruent with patients’ technological capacity to be most effective. Minimally technologically proficient patients at safety-net emergency departments may be candidates for future mHealth interventions, which can be optimized by selecting appropriate technological modalities: SMS text messages, instant messages, and, to a lesser extent, app-based modalities. Training strategies for minimally technologically proficient patients may help increase engagement and should be studied further. The inability to use text messages was the most common of the technological ineligibility criteria. This presents an opportunity to examine the role of basic mobile training or subsidized cellular connectivity to increase intervention effectiveness in this patient population. Technological proficiencies have improved over the course of successive Pew National Survey of Latinos Mobile Usage Surveys [2], as well in surveys at our own institution [22]. Institutional stakeholders interested in mHealth interventions for patient populations at safety-net emergency departments should periodically survey patients technological capacities to determine which technological modality to use.

Future emergency department–based mHealth interventions should embrace the diversity encountered at safety-net emergency departments as most demographic characteristics among our participants were not associated with classification as highly technologically proficient. Moreover, recruiting diverse patient populations to clinical trials can highlight nuances in intervention effectiveness in racial and ethnic subgroups [29,30]. We were able to capture different characteristics (race and ethnicity, country of birth, and primary languages spoken) than those of the national population. Resource-limited emergency departments serving minority and historically underserved patient populations should be considered uniquely suited for mHealth interventions. These emergency department patients can improve the design of mHealth trials by increasing ethnic and socioeconomic diversity among participants. We encourage other mHealth researchers to consider safety-net emergency departments as viable clinical settings to recruit a patient cohort with different demographic characteristics.

### Limitations

There are limitations to our analysis. The study was conducted at a single medical center. Multicenter studies are needed to understand the full scope of technological proficiencies nationally. However, this institution allowed us to focus on an understudied patient cohort recruited directly from a busy urban safety-net emergency department. Our cohort also had an overrepresentation of patients of Hispanic and Latino ethnicity. Future studies should include centers with more diverse representation. We only collected detailed demographic and mobile phone use information from patients enrolled in the mHealth intervention; therefore, we were unable to analyze all screened patients in the technology proficiency latent class analysis. Additionally, technological proficiency was self-reported introducing potential information bias, but it allowed us to examine what patients believed they were capable of, which may be a better indicator of modalities they will adopt. We did not offer mobile technology training to patients, which may have impacted engagement metrics. Future studies are needed to examine the costs and benefits of training patients in unfamiliar modalities.

### Conclusions

Emergency department–based mHealth interventions should allow institutional stakeholders to take advantage of costly unscheduled emergency care to engage patients in chronic disease management. Safety-net emergency departments are feasible for mHealth interventions; in our study, nearly one-fifth of emergency department patients with diabetes were equipped with the technological ability and access to participate in SMS text message–based interventions. Future intervention developers should consider the age and level of education of participants as they may be associated with variations in mHealth engagement, as found in our latent class analysis.

## Abbreviations

mHealth: mobile health
